# Hyperpolarized ^13^C‐MRS can Quantify Lactate Production and Oxidative PDH Flux in Murine Skeletal Muscle During Exercise

**DOI:** 10.1002/nbm.70020

**Published:** 2025-04-02

**Authors:** M. Kate Curtis, Jordan J. McGing, Brianna J. Stubbs, Vicky Ball, Lowri E. Cochlin, David P. O'Neill, Christoffer Laustsen, Mark A. Cole, Peter A. Robbins, Damian J. Tyler, Jack J. Miller

**Affiliations:** ^1^ Department of Physiology, Anatomy and Genetics University of Oxford Oxford UK; ^2^ Oxford Centre for Clinical Magnetic Resonance Research Oxford UK; ^3^ Buck Institute for Research on Aging Novato California USA; ^4^ Department of Biomedical Engineering, McCormick School of Engineering and Applied Science Northwestern University Evanston Illinois USA; ^5^ The MR Research Centre, Health Aarhus University Aarhus Denmark; ^6^ University of Nottingham Medical School Nottingham UK

**Keywords:** ^13^C MRS, hyperpolarized ^13^C, magnetic resonance, mouse models, muscle metabolism

## Abstract

Existing techniques for the non‐invasive in vivo study of dynamic changes in skeletal muscle metabolism are subject to several limitations, for example, poor signal‐to‐noise ratios which result in long scan times and low temporal resolution. Hyperpolarized [1‐^13^C]pyruvate magnetic resonance spectroscopy (HP‐MRS) allows the real‐time visualization of in vivo metabolic processes and has been used extensively to study cardiac metabolism, but has not resolved oxidative phosphorylation in contracting skeletal muscle. Combining HP‐MRS with an in vivo muscle hindlimb electrical stimulation protocol that modelled voluntary exercise to exhaustion allows the simultaneous real‐time assessment of both metabolism and function. The aim of this work was to validate the sensitivity of the method by assessing pyruvate dehydrogenase (PDH) flux in resting vs. working muscle: measuring the production of bicarbonate (H^13^CO_3_^−^), a byproduct of the PDH‐catalysed conversion of [1‐^13^C]pyruvate to acetyl‐CoA. Mice (*n* = 6) underwent two hyperpolarized [1‐^13^C]pyruvate injections with ^13^C MR spectra obtained from the gastrocnemius muscle to measure conversion of pyruvate to lactate and bicarbonate, one before the stimulation protocol with the muscle in a resting state and one during the stimulation protocol. The muscle force generated during stimulation was also measured, and ^13^C MRS undertaken at a point of ~50% fatigue. We observed an increase in the bicarbonate/pyruvate ratio by a factor of ~1.5×, in the lactate/pyruvate ratio of ~2.7×, together with an increase in total carbon (~1.5×) that we attribute to perfusion. This demonstrates profound differences in metabolism between the resting and exercising states. These data therefore serve as preliminary evidence that hyperpolarized ^13^C MRS is an effective in vivo probe of PDH flux in exercising skeletal muscle and could be used in future studies to examine changes in muscle metabolism in states of disease and altered nutrition.

## Graphical abstract

This validated combining hyperpolarized [1‐^13^C]pyruvate MRS with in vivo muscle stimulation to assess skeletal muscle metabolism during exercise in real time. Mice underwent MRS pre/post gastrocnemius stimulation, measuring conversion of [1‐^13^C]pyruvate to lactate and bicarbonate. Significant increases in ^13^C incorporation into bicarbonate and lactate were observed during exercise vers. This method effectively distinguishes metabolic flux between resting and exercising states, providing a novel tool for investigating skeletal muscle metabolism in vivo.

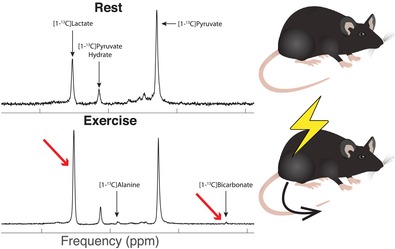

**Abbreviations**
ALT: alanine transaminase
ATP: adenosine triphosphate
CA: carbonic anhydrase
CK: creatine kinase
CoA: coenzyme A
CPT1: carnitine palmitoyltransferase I
CSA: cross‐sectional area
DCA: dichloroacetate
ECG: electrocardiogram
FOV: field of view
HP‐MRS: hyperpolarized magnetic resonance spectroscopy
LDH: lactate dehydrogenase
MRI: magnetic resonance imaging
MRS: magnetic resonance spectroscopy
PCr: phosphocreatine
PDH: pyruvate dehydrogenase
SNR: signal‐to‐noise ratio
STIM: stimulation
TCA: tricarboxylic acid
TE: echo time
TR: repetition time
VO_2_ peak: peak oxygen uptake
W_max_
: maximum power output

## 1 Introduction

Several Magnetic Resonance Imaging and Spectroscopy (MRI/MRS) techniques enable the assessment of skeletal muscle metabolism and offer a non‐invasive alternative to biological sampling and the use of biochemical analysis methods such as stable, nonradioactive isotope tracers. ^31^P MRS enables quantification of high energy phosphate concentrations [1], their metabolism during exercise and recovery [2] and metabolite exchange kinetics under steady state conditions (i.e., measuring creatine kinase (CK) kinetics via saturation transfer experiments [3]). These ^31^P MRS readouts correlate well with tissue level measurements of muscle mitochondrial respiration during biochemical assessment [4], providing that the exercise stimulus does not evoke significant intracellular acidosis [5, 6]. This enables inferences to be made on muscle mitochondrial function albeit during recovery from a relatively narrow range of low to moderate exercise intensities [7, 8].

Similarly, ^1^H MRS enables the quantification of skeletal muscle lactate concentrations [9, 10] as well as myocellular lipid concentrations. Lactate is a key intermediary metabolite reflecting substrate level phosphorylation, with roles as a fuel [11] and in cell signalling [12]. However, attempts to measure muscle lactate concentrations during ^1^H MRS experiments are limited by the inherently low signal‐to‐noise ratio (SNR) of lactate, which is present in millimolar concentrations in skeletal muscle, and the fact that multiple points of regulation means that the effective absolute lactate concentration can change far less rapidly than changes in metabolic fluxes, meaning that nearly 200 years after its discovery, the role of lactate in exercising skeletal muscle is still of scientific interest and debate [13]. Overlapping lipid peaks at clinical field strengths further impedes accurate lactate quantification via ^1^H‐MRS, requiring advanced techniques such as spectral editing. This limits the temporal resolution of accurate muscle lactate measurements that can be achieved with ^1^H‐MRS [14] and can make ^1^H MRS acquisitions highly technically challenging [15]. This imposes practical limitations on in vivo studies. Collectively these limitations restrict the use of ^1^H MRS to the quantification of steady‐state muscle lactate concentrations, which does not reflect the dynamic changes in lactate which occur during exercise and recovery and which may reveal key information on muscle metabolic function.

^13^C MRS isotope enrichment offers a further non‐invasive window into muscle metabolism in vivo. The combined use of ^13^C glucose and ^13^C acetate infusions during ^13^C MRS enables quantitation of TCA cycle activity and, in tandem with ^31^P MRS, has been used to characterise decreased TCA cycle flux and ATP synthesis in human ageing [16]. However, such experiments remain challenging, owing to the requirement for ^13^C substrate infusion, repeated biological sampling, multi‐nuclear hardware capabilities, and the poor temporal resolution of ^13^C MRS data (~10‐minutes [17]) due to the inherently low SNR of ^13^C labelled substrates and metabolites.

Hyperpolarized [1‐^13^C]pyruvate MRS overcomes the SNR limitation of ^13^C MRS and represents a relatively novel imaging modality capable of directly quantifying real‐time PDH flux (which has a sufficiently large ∆G to be irreversible) and the metabolic products of potentially reversible exchange reactions to [1‐^13^C]lactate and [1‐^13^C]alanine in vivo at a temporal resolution of 2–4 s [18]. As the product of glycolysis, pyruvate resides at a central point of several key metabolic pathways involved in the rapid provision of ATP during exercise. The enzyme complex pyruvate dehydrogenase (PDH) catalyses the conversion of glycolytically‐derived pyruvate into acetyl‐CoA and represents a fundamental regulatory step that determines the relative contribution of carbon from either glycolysis or beta oxidation of fatty acids to the TCA cycle. This decarboxylation of ^13^C labelled pyruvate and the resulting CO_2_ production, which rapidly exchanges with the bicarbonate pool via carbonic anhydrase (CA), enables quantification of oxidative phosphorylation in vivo. Due to its central role in metabolism, PDH activity is tightly regulated in vivo [19]. Acute changes in PDH activity occur in response to changes in intracellular conditions (i.e., concentrations of Ca^2+^, ATP and acetyl‐CoA), with increasing PDH activity seen during increasing exercise intensity [20]. However, activity can also be can be altered chronically by factors, such as genetic deficiencies [21], disease [22], exercise training [23], as well as nutritional [24, 25] and pharmacological interventions such as dichloroacetate (DCA) administration [26]. The role of lactate and alanine metabolism in muscle metabolism is more complex: whilst both lactate [27] and alanine [28] are excreted by muscles undergoing short, high intensity exercise, skeletal muscle can both consume and produce lactate either at rest or through longer duration periods of steady‐state exercise [29]. There is evidence of the small‐scale spatial transport of lactate between consuming and producing cells in mixed muscle groups during exercise [30]; in highly intense exercise lactate efflux is maximal and limited by monocarboxylate transporter expression and perfusion [31]. Therefore, during short‐duration intense exercise, the hyperpolarized [1‐^13^C]pyruvate experiment is able to uniquely probe the degree of label exchange into [1‐^13^C]lactate and [1‐^13^C]alanine, and from the former form a proxy for total glycolytic flux.

Temporal changes in muscle metabolism in vivo measured using ^31^P MRS have been previously reported in a murine hindlimb preparation, with muscle contraction elicited by electrical stimulation of the sciatic nerve in low frequency continuous pulses [32], or via intermittent trains of medium frequency stimulation [33], the latter analogous to locomotory muscle contraction. The stimulation protocol in the latter study was designed to produce progressive, reproducible muscle fatigue without irreversible muscle damage or failure of action potential transmission. Similar hindlimb preparations using short‐duration stimulation protocols have since reported changes in muscle metabolism using [1‐^13^C]pyruvate MRS, showing an increase in the [1‐^13^C]pyruvate to [1‐^13^C]lactate ratio following 30 s of electrical stimulation [34]. In the same hindlimb preparation, a novel hyperpolarised multi‐bolus paradigm combined with spectroscopic imaging, characterised muscle metabolic response to a range of muscle contraction intensities. Glycolytic flux increased with muscle contraction and was reflected in the amplitude of the ^13^C lactate signal. This signal was proportional to the intensity of muscle stimulation and specific to fast twitch muscle fibres [35]. More recently, ^13^C pyruvate MRI/MRS has been translated for use on healthy human volunteers. The conversion of ^13^C pyruvate to ^13^C lactate and ^13^C bicarbonate increased during voluntary repeated plantar flexion exercise using a resistance band, reflecting an increased rate of label exchange of pyruvate through LDH and PDH during exercise [36].

The aim of the current work was to develop and validate the in vivo murine preparation previously described by Cole et al. [33] that models intense voluntary exercise in order to assess sustained skeletal muscle function combined with real‐time assessment of ^13^C pyruvate metabolism to [1‐^13^C]lactate and ^13^C bicarbonate. Once validated, this technique would be beneficial in determining previously unquantifiable temporal changes in metabolic fluxes resulting from genetics [21], disease [22], exercise training [23], and pharmacological intervention [26, 37]. Whilst the hyperpolarised ^13^C MRS technique can be used to study other enzymatic pathways via injecting alternative hyperpolarized substrates [38], PDH was considered to be an ideal target to validate this technique, given its central role in exercise metabolism and substrate utilisation, especially when combined with the favourable nuclear properties of [1‐^13^C]pyruvate which make it amenable to DNP.

## 2 Methods

**FIGURE 1:**
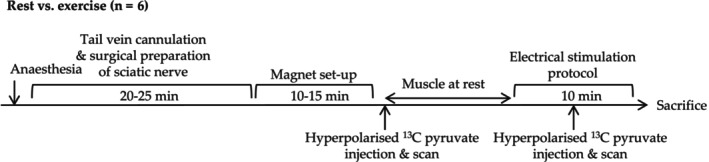
Schematic illustration of the overall study design. Animals underwent two hyperpolarized [1‐^13^C]‐pyruvate injections; one at rest and one during electrical stimulation/steady‐state gastrocnemius contraction.

Three‐month‐old C57BL/6 male mice (Envigo, UK) underwent an in vivo gastrocnemius muscle stimulation protocol in conjunction with two hyperpolarized [1‐^13^C]pyruvate injections, one before the stimulation protocol with the muscle in a resting state and one during the stimulation protocol (Figure 1). All animal procedures were compliant with both the UK Home Office Guidance on the Operation of the Animals (Scientific Procedures) Act 1986 and the University of Oxford Animal Ethics Review Committee. Animals had free access to standard rodent chow and water. Experiments were performed using a horizontal bore Agilent 7 T MRI system (DDR console, Agilent, Santa Clara, USA). Dynamic nuclear polarisation was performed using a previously described prototype hyperpolarizer [39]. All compounds were obtained from Sigma Aldrich (Gillingham, Dorset, UK) unless otherwise stated.

### 2.1 Surgical Preparation

**FIGURE 2:**
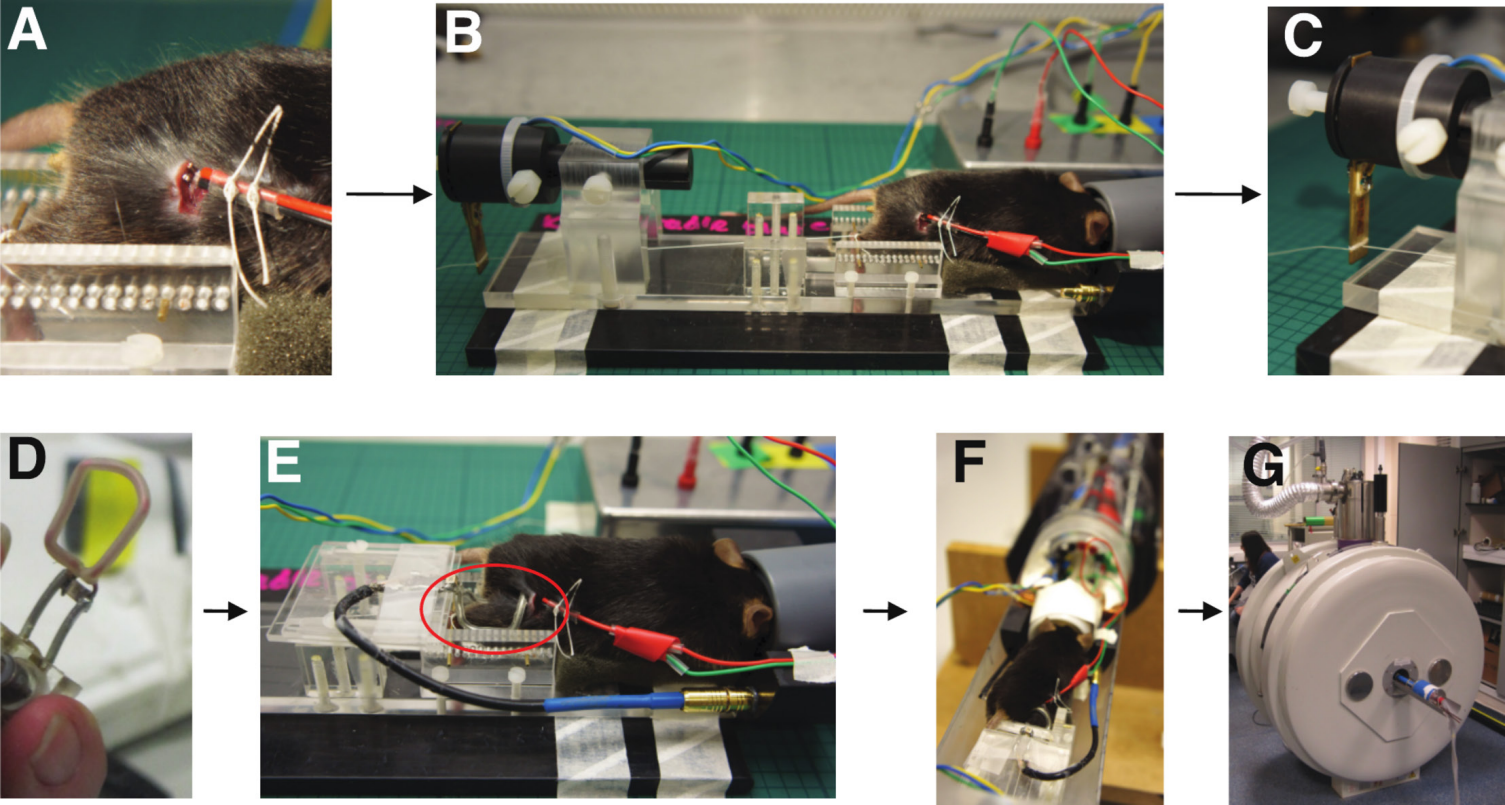
Experimental set up for investigating skeletal muscle metabolism using hyperpolarized [1‐^13^C] pyruvate. (A) Electrodes placed around the sciatic nerve. (B and C) Knee and ankle joints immobilized. Suture thread tied around the calcaneal tendon and attached to a force transducer. (D and E) Home‐built ^13^C saddle‐shaped RF surface coil placed over the gastrocnemius muscle. (F and G) Mouse placed in a horizontal Agilent 7 T MRI system.

Mice were anaesthetized using 2.5%–3% isoflurane in O_2_ and N_2_O (1.8 L/min:0.2 L/min) and maintained at 2% isoflurane via a nose cone during the experiment. A cannula was inserted into the tail vein for intravenous injection of hyperpolarized [1‐^13^C]pyruvate solution. The sciatic nerve was exposed via blunt dissection, and electrodes were sutured in place distal to the tibial nerve branch (Figure 2A). Electrodes were connected to a Digimeter DS7 stimulus isolator which delivered a constant current stimulus and which in turn was connected to a PowerLab analogue‐to‐digital converter system (ADInstruments Ltd) to record both stimulation train timings and muscular developed force, as described later. The mouse was placed in a bespoke Perspex cradle, designed and manufactured for this protocol and the knee and ankle joints were immobilized (Figure 2B) before the calcaneal tendon was attached to an isometric NMR‐compatible strain gauge force transducer via a suture thread (Figure 2C) that could be externally tensioned. A bespoke 10 mm ^13^C saddle‐shaped RF surface coil was placed over the gastrocnemius muscle (Figure 2D,E). Air heating‐maintained body temperature and ECG was monitored throughout the experiment via electrodes placed subcutaneously.

### 2.2 Hyperpolarized ^13^C MRS

Correct positioning of the hindlimb at the centre of the scanner was confirmed by the acquisition of an axial proton FLASH localiser image (TR/TE, 2.33/1.17 ms; matrix size, 64 × 64; field of view (FOV), 60 × 60 mm^2^; slice thickness, 2.5 mm; excitation flip angle, 15°). An ECG‐gated slab‐selective shim (15 kHz bandwidth, 5° flip angle, 14 mm thick) was used to reduce the proton linewidth to better than 0.5 ppm. [1‐^13^C]pyruvic acid was hyperpolarized and dissolved and neutralised as previously described [18, 40]. An aliquot of 0.2 mL of 80 mM hyperpolarized [1‐^13^C]pyruvate solution was injected over 10 s via the previously placed tail vein cannula. With the muscle in a resting state, spectra were acquired for one‐minute post‐injection, using a 10 μs 15° hard excitation pulse (TR = 1 s, 8 kHz bandwidth), with signal localised to the gastrocnemius muscle through the surface coil used. The first 30 spectra after the appearance of the pyruvate peak were summed and analysed using the AMARES algorithm in the jMRUI software package [41] and results shown as the ratio of the returned amplitude of the metabolite of interest to that of pyruvate.

### 2.3 In Vivo Gastrocnemius Muscle Stimulation

Thirty minutes after spectra were acquired from resting muscle, the stimulation protocol began. Isometric force production of the hindlimb muscles was measured using a PowerLab computer system connected to the force transducer previously calibrated by a series of small test masses under the assumption that acceleration due to gravity, *g* = 9.81 m/s^2^. Resting tension of the calcaneal suture was set at 30 cN, and stimulation intensity was set by evoking a series of twitch contractions via single 100 μs duration stimuli, increasing in 10 mA steps, and monitoring force production. The current where no further increase in force was observed was considered the maximal stimulation level, typically 60 mA.

Subsequently, to mimic exercise, a previously‐validated murine stimulation protocol [33] consisting of intermittent trains of eight pulses of 100 μs at 30 Hz followed by a rest periods of 1.25 s was repeated over a 10‐min period, and resulting force production recorded over time. This intermediate frequency stimulation protocol evoked semi‐fused tetanic contractions, in which the muscle as a whole undergoes a partially smooth, sustained contraction with minimal but present oscillations in tension between each pulse. This protocol, adapted from a protocol designed to delineate the fatigue characteristics of individual motor units [42, 43], avoids loss of neuromuscular transmission. The resulting skeletal muscle fatigue thus reflected impairment of skeletal muscle cell function, whilst intermittently inducing physiologically relevant ischaemia with each tetanic contraction [44].

**FIGURE 3:**
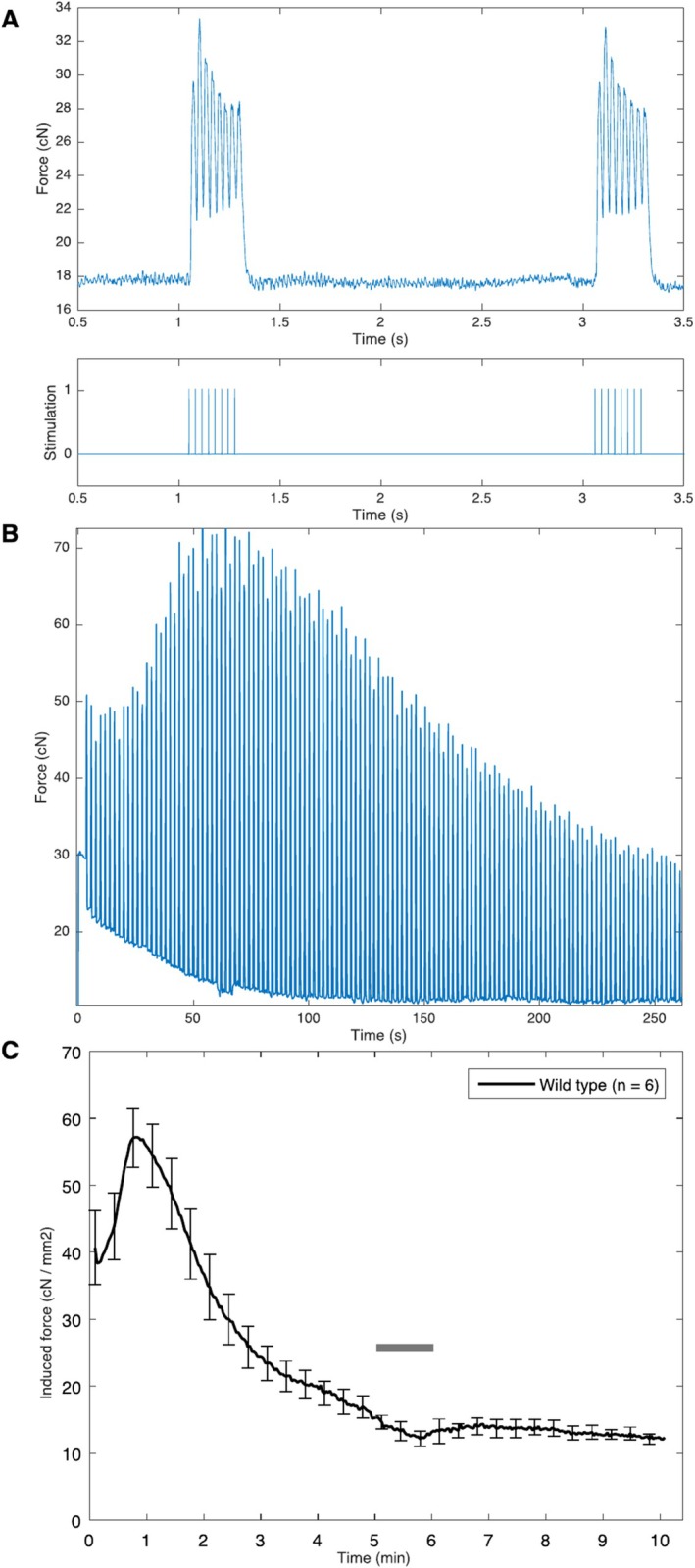
(A) The bottom trace shows two intermittent stimulation trains, each consisting of eight pulses of 100 μs at 30 Hz followed by a 1.25 rest. Three hundred pulse trains were applied over 10 min. The top trace displays the semi‐fused tetanic contractions produced in response to the stimuli over a short timescale. (B) The contractile force trace produced by one mouse over a subsection of the 10‐min stimulation period, clearly showing the baseline force and stimulated force as measured prior to baseline correction and normalisation via CSA. (C) A graph showing the induced force production (i.e., the baseline‐corrected force) over the 10‐min exercise period with error bars denoting SEM for each animal considered. Force produced is corrected to the cross‐sectional area (CSA) of the hindlimb. The grey bar represents the time during which hyperpolarized [1‐^13^C]pyruvate solution was injected and spectra obtained.

After an initial period of stabilisation to enable the muscle to reach a metabolic steady state corresponding to sustained exercise, hyperpolarized [1‐^13^C]pyruvate was again injected in to the tail vein and spectra acquired during exercise. High‐resolution gradient echo ^1^H images (TR/TE 121.5/4.4 ms, 14 slices, 50 × 50 mm FOV, 0.8 mm thick, 192 × 192 matrix, 20° FA), analysed in Fiji/ImageJ, were used to obtain the cross‐sectional area (CSA) of the hindlimb muscles. Data from semi‐fused tetanic contractions were analysed with a bespoke MATLAB peak‐finding script that accounted for variations in baseline tension. For each of the 300‐pulse long pulse trains, we calculated the difference between the peak tensile force of a stimulated twitch and the preceding baseline tension. We define this measurement as the induced force production, and normalised this value to muscle size by dividing by the CSA of the hindlimb. Mean baseline tension, peak tension and induced force production were plotted over time with together with the illustrative standard error on the mean shown every 20 s (Figure 3).

### 2.4 Statistical Methods

Data were analysed using R version 4.2.3. All summary statistics are given as mean ± standard error. Comparisons of the ^13^C metabolites between the resting state and on exercise were assessed using paired Student's *t*‐tests with Tukey's ‘Honestly significant differences’ method of multiplicity correction, under the assumption that the error in the denominator of the ratio (pyruvate) is small and therefore data are normally distributed [45]; for the case of the ratio of lactate to bicarbonate (where this assumption is not true) a (paired) Wilcoxon test was used. Statistical significance was considered at *p* < 0.05.

## 3 Results

We were able to undertake measurement of in vivo skeletal muscle metabolites combined with force generation in real‐time. Electrical stimulation caused contraction and an increase in the observed tension in the suture immediately following stimulation, as shown in Figure 3A. Over a longer period, the measured force initially increased before decaying due to exhaustion, together with a shift in the baseline reading. The baseline‐corrected induced force generated over the 10‐min duration of the experiment, as illustrated in Figure 3B consistently demonstrated a reduction of greater than 50% in contractile force over the exercise period (Figure 3C), corresponding to fatigue. Force generation over the 10‐min stimulation period, corrected for CSA of the hindlimb, calculated from baseline tension and peak tension is shown in Figure 3C. Reductions of contractile force in excess of 50% are exhibited over the 10‐min exercise period.

**FIGURE 4:**
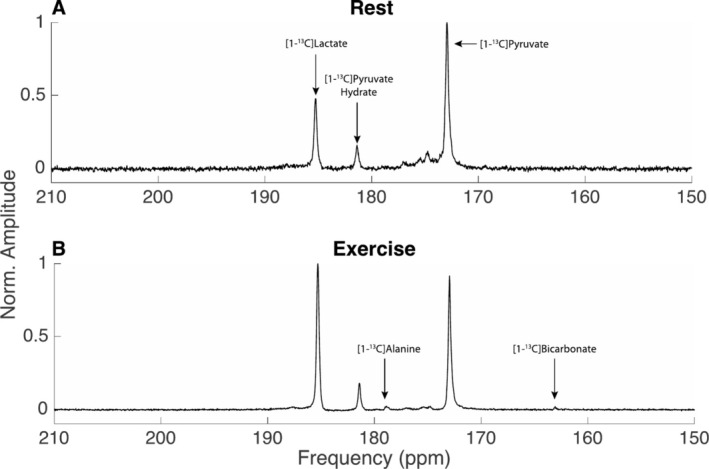
Representative ^13^C spectra acquired at rest (A) and during steady‐state muscle contraction (B), together with metabolite peaks detected and quantified. Spectra were referenced to [1‐^13^C]pyruvate. A flip angle calibration was carried out on a point phantom at the depth of the target muscle within the saddle coil for these acquisitions.

**FIGURE 5:**
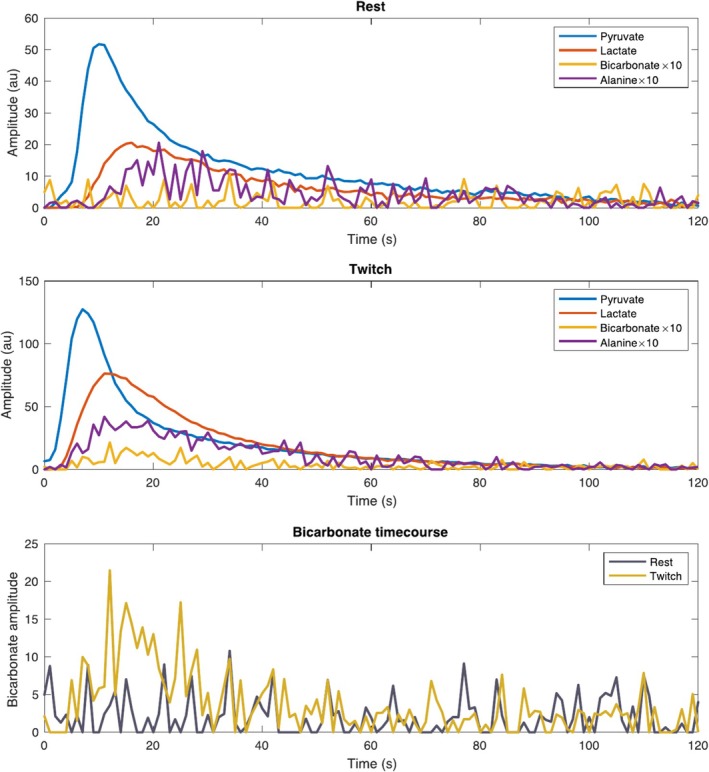
Representative pyruvate and lactate timecourses shown for rest and exercise conditions, after quantification by AMARES. Bicarbonate quantification over time was limited by poor spectral SNR in the resting state, but more possible in the exercising state. Note that both bicarbonate and alanine have been multiplied by a factor of 10 compared to pyruvate and lactate.

**FIGURE 6:**
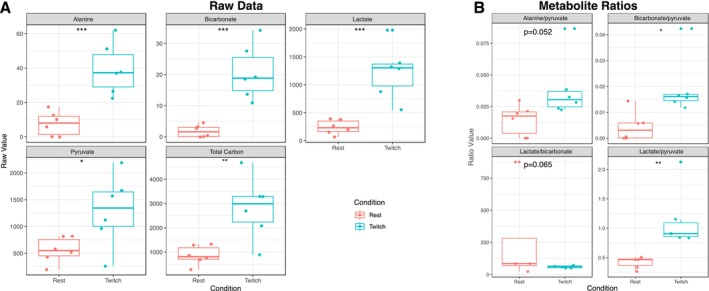
Quantification of temporally summed ^13^C spectra by the AMARES algorithm, either directly (A) or after obtaining the ratio of alanine, lactate and bicarbonate to pyruvate (B). * Significance was determined using a paired Student's two‐tailed *t*‐test at *p* < 0.05, with the exception of the lactate/bicarbonate ratio which was assessed using a paired Wilcoxon test.

The conversion of the [1‐^13^C]pyruvate to [1‐^13^C]lactate and ^13^C bicarbonate reflecting ^13^C label exchange into the existing intramyocellular lactate pool and muscle PDH flux was measured successfully at rest and during stimulation, over time (Figures 4 and 5). During stimulation, real‐time increases in the conversion of [1‐^13^C]pyruvate to [1‐^13^C]lactate and ^13^C bicarbonate were resolved; although it was possible to resolve metabolites dynamically with typical characteristic timecourses, bicarbonate quantification was not reliably possible in the rest condition in individual spectra, but quantification was possible after temporal summation of spectra, which, as expected, improved SNR substantially both at rest (Figure 4A) and during stimulation (Figure 4B). A significant increase in all quantified metabolites was detected between rest and stress, detailed in Table 1 and Figure 6A, together with an increase in the total amount of labelled carbon detected (i.e., their sum). An increased flux through pyruvate dehydrogenase in the muscle during exercise was demonstrated by a significant increase in [1‐^13^C] label incorporation from pyruvate through to bicarbonate when compared to muscle at rest (0.004 ± 0.006; 0.02 ± 0.011; respectively, *p =* 0.0146*)*. The conversion of [1‐^13^C]pyruvate, via lactate dehydrogenase, to [1‐^13^C]lactate was also significantly increased during exercise when compared to rest (0.417 ± 0.097; 1.128 ± 0.503; respectively, *p* = 0.0068) (Figure 6B). We computed the ratio of lactate to bicarbonate in an attempt to more specifically reflect the mitochondrial partitioning of pyruvate metabolism irrespective of total pyruvate delivery and uptake. We found that this ratio was highly variable, particularly in the rest case where the ^13^C‐bicarbonate label was close to zero, and therefore expect the resulting distribution to be non‐normal analytically a priori. There was no significant difference between the two conditions via a paired Wilcoxon test (*p* = 0.065).

**TABLE 1:** Detailed summary statistics detailing mean and standard deviations for the *n* = 6 experiments performed; *p*‐values refer to those from a paired *t*‐test and are multiplicity adjusted by Tukey's HSD method, with the exception of the lactate/bicarbonate ratio, which was assessed utilising a paired Wilcoxon test owing to the extreme non‐normality of the low‐SNR ratio.

Metabolite	Rest $\bar{x}$	Rest s	Twitch $\bar{x}$	Twitch s	*p*	Significance
Alanine	7.653	6.993	39.483	14.952	0.0008	***
Alanine/pyruvate	0.014	0.012	0.039	0.024	0.0518	ns
Bicarbonate	1.826	1.947	20.700	8.764	0.0004	***
Bicarbonate/pyruvate	0.004	0.006	0.020	0.011	0.0146	*
Lactate	241.333	128.738	1235.000	486.331	0.0007	***
Lactate/pyruvate	0.417	0.097	1.128	0.503	0.0068	**
Pyruvate	559.667	238.359	1295.500	667.735	0.0293	*
Pyruvate Hydrate	51.633	32.787	224.867	127.523	0.0091	**
Lactate/Bicarbonate	267.018	406.55	60.23	8.827464	0.0651	ns
Total Carbon	866.703	393.466	2819.830	1278.757	0.0051	**

The ‘significance’ column is a summary of the *p*–value where ‘ns’ denotes *p* > 0.05, ‘*’ 0.01 < *p* < 0.05; ‘**’ 0.001 < *p* < 0.01 and ‘***’ denotes *p* < 10^−3^.

## 4 Discussion

This study investigated the combined use of [1‐^13^C]pyruvate MRS together with electrical stimulation to simultaneously probe skeletal muscle function and metabolism in vivo. Hyperpolarized [1‐^13^C]pyruvate MRS enabled resolution of [1‐^13^C]pyruvate metabolism to [1‐^13^C]lactate, [1‐^13^C]alanine and ^13^C bicarbonate at rest and during electrical stimulation of murine gastrocnemius muscle. In the resting state spectra, [1‐^13^C]lactate was clearly resolved (Figure 4 and Figure 5A). Furthermore, hyperpolarized [1‐^13^C]pyruvate MRS was able to delineate real‐time increases in label exchange through LDH and oxidative phosphorylation during exercise relative to rest. This was reflected by a simultaneous increase in the conversion of [1‐^13^C]pyruvate to [1‐^13^C]lactate and ^13^C bicarbonate during steady‐state electrical stimulation.

These data corroborate previous [1‐^13^C]pyruvate studies, which demonstrated elevated glycolysis during exercise in rodent hindlimb muscles [34, 35], as shown by an increase in ^13^C‐lactate signal. Additionally, we demonstrated the feasibility of measuring muscle mitochondrial metabolism during electrical stimulation by quantifying the ^13^C‐bicarbonate signal produced during the decarboxylation of [1‐^13^C]pyruvate. Although it is usually the case that the inherent variability in the resultant polarization provided through hyperpolarization techniques means that quantifying raw peak intensities is arguably of questionable value, we observed a consistent increase in both total carbon signal and, as the noise floor is approximately constant for a small coil‐noise dominated saddle coil, therefore spectral SNR. This is of further interest, as this increase on exercise relative to rest we hypothesise is attributable to increased muscle perfusion directly – in health well matched to metabolism, but frequently dysregulated in pathology. We were not able to directly quantify muscle perfusion within the necessarily short time course of these experiments, but note that in healthy muscle a number of mechanisms exist to increase muscle perfusion and that both it and metabolism are likely in a steady state and stable during the time course of the ^13^C acquisition [33]. However, although present during exercise, bicarbonate production was difficult to observe in time‐resolved spectra obtained at rest and we therefore elected to quantify spectra via the ratio of quantified peaks in temporally integrated data, producing peak ratios that are known to correlate with specific rate constants determined by first‐order metabolic models in vivo, typically with *R* ^2^ >  0.9 [46, 47].

During exercise several energy systems are simultaneously activated to meet the large increase in muscle ATP demand. At the onset of aerobic exercise and during high intensity anaerobic contraction, the increased ATP demand is met primarily by substrate level phosphorylation (i.e., PCr turnover and anaerobic glycolysis) owing to the ability of these control systems to facilitate rapid ATP supply at a millisecond level [48]. The significant increases in ^13^C‐lactate to ^13^C‐pyruvate ratio seen here (0.417 ± 0.097;1.128 ± 0.503, *p* = 0.0068) reflects increased muscle glycolytic flux and conversion of [1‐^13^C]pyruvate to [1‐^13^C]lactate during electrical stimulation which is consistent with earlier ^13^C‐pyruvate MRS experiments: Leftin et al. demonstrated stepwise increases in the ratio of [1‐^13^C]lactate to total carbon following 15 and 30 s of electrical stimulation of the hindlimb muscle in rodents. This peaked on 30 s of stimulation and plateaued at 90 s [34], a set of time points that are associated with substantial changes in vasodilation and well within the ‘equilibration period’ of the dynamic response to intense exercise [33]. Further, the total [1‐^13^C]lactate to total carbon ratio was increased on voluntary plantar flexion exercise of an unknown relative intensity in healthy human volunteers compared to rest (0.18 ± 0.04;0.31 ± 0.02, *p* = 0.04) [36]. We argue that this increase in the lactate to pyruvate ratio denotes a specific metabolic shift despite increased pyruvate availability: were metabolism not increased concomitantly with the vasodilation during exercise, the pyruvate signal would increase as a result of increased perfusion and blood volume during exercise (of 50%–175% [49]) but lactate would not, as the supraphysiologic pyruvate bolus would not be limiting, yielding a decrease in the lactate to pyruvate ratio, rather than the observed increase. The elevations in ^13^C‐lactate to pyruvate ratio reported here and during earlier ^13^C‐pyruvate MRS studies is consistent with the activation of phosphorylase [50] and increased muscle glycolytic flux demonstrated by tissue level measurements resulting in increased flux of pyruvate through LDH and subsequent lactate production [51] across moderate to high exercise intensities due to the accumulation of pyruvate and NADH. The increase in tissue concentration of muscle lactate during submaximal aerobic exercise 55% W_max_ relative to rest in human subjects (6.3 ± 0.9 vs. 15.6 ± 3.4 mmol/kg dry mass, *p* < 0.05) was of a comparable magnitude to the increase in our ^13^C‐lactate to pyruvate ratio (0.417 ± 0.097 to 1.128 ± 0.503, *p* = 0.0068) during steady‐state electrical stimulation. Whilst glycolysis, alongside PCr depletion, is a predominant contributor to muscle ATP demand during high intensity contractions [48, 50, 52], simultaneous increases in PDH flux are observed alongside increased muscle glycolysis during exercise, both as a function of time during the later stages of repeated maximum‐intensity exercise [48, 52] and during steady‐state aerobic exercise [20, 50, 53]. Our electrical stimulation data were acquired at 5 min into a 10 min steady‐state contraction protocol and therefore required a significant contribution of aerobic metabolism to meet muscle ATP demand [54]. Indeed, the stimulation protocol evoked a substantial increase in ^13^C‐bicarbonate to [1‐^13^C]pyruvate (0.004 ± 0.006; 0.020 ± 0.011, *p* = 0.0146), which is consistent with the activation of PDH and the contribution of oxidative phosphorylation to muscle ATP demand during steady‐state aerobic exercise. Stepwise increases in PDH activation are observed during steady state cycling exercise in humans at intensities ranging from 30%–90% VO_2_ peak [20, 50, 53]. PDH activity is significantly higher at 75% W_max_ relative to rest (0.57 ± 0.08 vs. 1.67 ± 0.32 mmol, *p* < 0.05) which occurs alongside increased muscle lactate production (6.3 ± 0.9 vs. 29.5 ± 2.4 mmol/kg dry muscle *p* < 0.05) and degradation of muscle glycogen concentrations (526 ± 22 vs. 200 ± 31 mmol/kg dry muscle *p* < 0.05) [20]. Previous rodent exercise studies using [1‐^13^C]pyruvate MRS have not quantified muscle PDH flux and reported only ^13^C lactate production [34, 35]. However, these [1‐^13^C]pyruvate MRS data are congruent with biochemical assessment of muscle metabolic flux, demonstrating a simultaneous elevation of skeletal muscle glycolysis and PDH flux on prolonged, steady‐state contraction.

With further development this technique may offer a useful tool to further basic research on muscle metabolism in vivo [55]. Previous rodent experiments encompassing the combined use of [1‐^13^C]lactate and [2‐^13^C]pyruvate have enabled the characterisation of skeletal muscle lactate kinetics under resting conditions and during dichloroacetate infusion [38]. Further application of ^13^C‐pyruvate MRI/S and the simultaneous assessment of skeletal muscle function to relevant rodent models of disease and genetic modification [55] could help to elucidate how alterations of particular genes impact on skeletal muscle function and metabolism in the animal as a whole.

Looking toward clinical applications, the recent validation of [1‐^13^C]pyruvate MRI for use in studies of human muscle metabolism [36] opens up the possibility for an in depth metabolic assessment of muscle health, function and pathophysiology [56, 57]. This development extends the current range of clinically feasible imaging modalities available for integration into multiparametric imaging paradigms, enabling a comprehensive assessment of skeletal muscle phenotype within a clinically feasible time frame and in contrast to phosphorus spectroscopy alone, permits the direct quantification of PDH flux and exchange into lactate, providing a measure for the utilisation of fats as opposed to sugars. Such advancements could offer unprecedented insights into muscle metabolic decline in conditions where it is not known if this precedes or antecedes functional decline (such as sarcopenia), as well as the response to exercise [57] and pharmacological intervention [37].

## 5 Limitations

The stimulation protocol mimicked the cyclical contraction pattern the gastrocnemius muscle would undergo during locomotion [33] and may be considered an improvement on existing techniques which employ isolated muscle strips or homogenates. Furthermore, the surgical preparation is far simpler than the isolated perfused hindquarter [58] or other intact limb‐only preparations. However, there are several limitations to this study. Despite using a surface coil close to a surgically exposed exercising muscle, we have found bicarbonate difficult to routinely quantify at rest in anaesthetised murine skeletal muscle, although PDH flux was observed during exercise. We did not directly quantify perfusion simultaneously with metabolism owing to the technical challenges posed by these experiments. Whilst we believe this surgical and electrical stimulation protocol to be a better representation of in vivo exercise compared to existing techniques and are directly controllable in intensity, one should note the inherent discrepancies between electrically evoked stimulation during general anaesthesia and voluntary muscle exercise modes utilised in human studies.

Of relevance to this work is the potential confounding effects of anaesthesia on the measurement of muscle metabolism. This has been investigated in other organs commonly probed via hyperpolarised [1‐^13^C]pyruvate MRS, such as the brain, where the cerebral metabolism of [1‐^13^C]pyruvate is heavily dependent upon isoflurane dosage [59] and both the pyruvate‐to‐lactate and pyruvate‐to‐bicarbonate labelling rates are less during anaesthesia with isoflurane relative to the awake state [60]. The role of general anaesthesia on rodent skeletal muscle mitochondrial function is not well documented, in contrast to organs such as the brain and heart. However, general anaesthesia is known to inhibit mitochondrial substrate oxidation in human quadricep muscles [61]; and isoflurane has been shown to increase Ca^2+^ release from the sarcoplasmic reticulum in mouse skeletal muscle ex vivo [62] and to increase the skeletal muscle arterial diameter of rats by (26 ± 2%, *p* < 0.05) [63] both of which may modulate indirect effects on the metabolism of [1‐^13^C]pyruvate in skeletal muscle. Other authors have used either isoflurane [34, 35] or alternative anaesthetic combinations such as fentanyl/fluanisone i.p. [33] (wherein the level of observable fatigue is comparable to this work). Based on these collective findings, future rodent studies should seek to investigate the effect of anaesthesia on skeletal muscle mitochondrial function/metabolism of hyperpolarised ^13^C pyruvate to ascertain the potentially confounding effects on metabolism.

## 6 Conclusion

An in vivo method of hyperpolarized [1‐^13^C]pyruvate spectroscopy combined with gastrocnemius muscle stimulation was successfully established. This technique facilitated the simultaneous assessment of skeletal muscle metabolism and function in a model of voluntary exercise to exhaustion. Our results demonstrate that the technique is sensitive enough to characterise both glycolytic and oxidative metabolism at rest and during electrically stimulated, steady state contraction. This technique may provide new in vivo metabolic insights into muscle decline in the numerous conditions for which mouse models are readily available.

## Conflicts of Interest

MKC and PAR received a research grant from Vifor Pharma, which did not have any intellectual input or other control over this study.

## Data Availability

The data that support the findings of this study are available on request from the corresponding author. The data are not publicly available due to privacy or ethical restrictions.
